# Supplementary material for: A common molecular logic determines embryonic stem cell self‐renewal and reprogramming
Source: EMBO J. 2018 Nov 27;38(1):e100003. doi: 10.15252/embj.2018100003 (PMC6316172; doi:10.15252/embj.2018100003)
Supplement: Supplementary file 1 — Appendix [file EMBJ-38-e100003-s001.pdf]

Summary of Appendix for

Elucidating a Biological Program for Resetting Pluripotent Identity

Content

1) Supplementary text related to Appendix Figure S8……………………………………………………..1

2) Appendix Supplementary Figures……………………………………………………….…………………………..3

Appendix Fig S1: (Related to Fig 1) Deriving and constraining the 0.832 ABN.

Appendix Fig S2: (Related to Fig 1) Predicting the relative potency of single factor forced expression in enhancing resetting to the naïve state.

Appendix Fig S3: (Related to Fig 3) Investigating dual factor expression in OEC2-GY118 EpiSCs.

Appendix Fig S4: (Related to Fig 3 and Fig 4A-B) A comparison of the resetting kinetics under empty vector control and dual factor expression, visualised on the 0.782 cABN.

Appendix Fig S5: (Related to Fig 4 and 5): Analysis of resetting time course using SPADE and clones generated by forced expression of Esrrb-T2A-Klf4 for 4 days.

Appendix Fig S6: (Related to Fig 6) Klf2 and Klf4 KO EpiSC generation and transgene free Klf2KO iPSCs.

Appendix Fig S7: (Related to Fig 7) Investigation of stat3 downstream effectors in EpiSC resetting (a-c, related to Fig 6), and LIF requirement in MEF reprogramming.

Appendix Fig S8: Investigation of the minimal model from the 0.717 cABN, which is simulated under an asynchronous update scheme.

3) Appendix Supplementary Tables………………………………………………………………………………….19

Appendix Table S1: siRNAs used in this study.

Appendix Table S2: Real-time quantitative PCR primers and probes.

Appendix Table S3**:** DNA oligonucleotides used to generate gRNAs and genotype Klf2 and klf4 knockouts.

Appendix Table S4: Custom Taqman OpenArray real-time quantitative PCR IDs used for the single cell gene expression.

1) Supplementary text related to Appendix Figure S8

Asynchronous Simulations

Here we compare the results of our methodology with the approach of simulating a single model under an asynchronous update scheme. For this investigation, we selected the minimal model from the 0.717 cABN (the network with the fewest interactions), which is shown in Fig. S8a. We ran simulations of this model using BooleanNet, a freely available Boolean network simulator [1], and examined the resetting dynamics produced under different network perturbations. For each experiment described below, 10000 independent simulations were run under an asynchronous update scheme in which one component is chosen at random to update at each step. We set the initial state to be equal to the GOF18 EpiSC state following discretisation (Fig. S1f).

First, we investigated the effect of single factor forced expression in EpiSC resetting. We calculated the proportion of simulations that reached the naïve state, and then determined the average step at which the naïve state was reached. This enabled us to compare the relative potency of each single factor overexpression to Control (2i+LIF only with not overexpression) (Fig. S8b), and to evaluate model predictions against experimental results (Fig. 1e). The model predicted that forced expression of any of Nanog, Sox2, Tbx3, Sall4, Esrrb, Klf2 and Klf4 would guarantee that the naïve state be reached. These perturbations subsequently differed in the average trajectory length to the naïve state. As for the analysis run for the cABN, Klf2, Klf4 and Esrrb emerged as the most potent inducers of resetting with the lowest number of average steps required to reach the naïve state. In contrast, Sall4, Sox2, and Oct4 were incorrectly predicted to enhance resetting. Finally, forced expression of Gbx2 and Stat3 were predicted to marginally increase the number of simulations reaching the naïve state, or to decrease the number of steps required to reach the naïve state, respectively. Only for Gbx2 could we observe experimentally a mild yet significant increase in colony number (Fig. 1e), thus we conclude that overall, 7 out of 11 predictions are supported by experiment, as the model incorrectly predicts the effect of Stat3, Sall4, Sox2, and Oct4.

We were able to interrogate these simulations further to examine gene activation kinetics by calculating the number of steps taken for each gene to reach a stable average expression across all simulations, which indicates the relative order of gene activation towards the naïve state (Fig. S8c). In the control case only a fraction of the simulations reached the naïve state, therefore when we calculated the Average Expression of each factor across all simulations we observed, for instance, that Esrrb expression reaches ~0.8. Conversely, factors like Gbx2 appear to activate in all simulations, also those not reaching the naïve state. Forced expression of Klf4 allows all simulations to reach the naïve state, and indeed the average expression of all factors is 1. Moreover, a stable state is reached more rapidly in the case of Klf4 forced expression, as indicated by the red lines on the right panels. Compared with the experimental data (Fig. 2b), in the control case, the model incorrectly predicts only the early activation of Gbx2 and agrees with the kinetics of all other factors investigated.

We similarly investigated the effect of single factor knockdown in EpiSC resetting. Using the number of simulations that reach the naïve state as a measure (Fig. S8d), the model predicts that the removal of Gbx2 and Tfcp2l1 do not have effect on EpiSC resetting, that removal of Klf4, Tbx3 and Sall4 had a significant partial effect on resetting efficiency, while Nanog, Esrrb, Oct4, Stat3, Sox2 and Klf2 are required for resetting. When compared to the experimental results (Fig. 6b), only Klf4 was incorrectly predicted.

Finally, we investigated dual factor forced expression in EpiSC resetting (Fig. S8e). We tested whether combinations of factors would have a synergistic effect over single factors alone using the average number of steps required to reach the naïve state as a measure (note that in all cases, all simulations reach the naïve state). Compared to our experimental results (Fig. 3a, right panel), the model incorrectly predicts that Klf4/Tbx3 together are no more efficient than either factor alone, and that Esrrb/Tfcp2l1 together demonstrate an additive effect, as shown by a reduction in the average number of steps required to reach the naïve state. Overall, 5 out of 7 predictions are supported experimentally (Fig. S8e, lower panel).

The set of predictions made by 0.717 minimal model under an asynchronous update scheme were compared to those generated from the 0.717 cABN (Fig. S8f). Overall, the minimal model demonstrates a high predictive accuracy of 75.86% across this set of tests, but this accuracy is lower than the predictive accuracy of the 0.717 cABN, which is 84.92%. Based on this comprehensive comparison, we conclude that 0.717 cABN captures models with behaviours relevant to the real biological process with individual models possessing high level of predictive accuracy. This also highlights the utility of our cABN approach in identifying candidate network models that can be subsequently used for simulation-based investigation, and undoubtedly can be further refined based on experiments.

References

1. Albert I, Thakar J, Li S, et al (2008) Boolean network simulations for life scientists. Source Code Biol Med 3:16. doi: 10.1186/1751-0473-3-16

2) Appendix Supplementary Figures

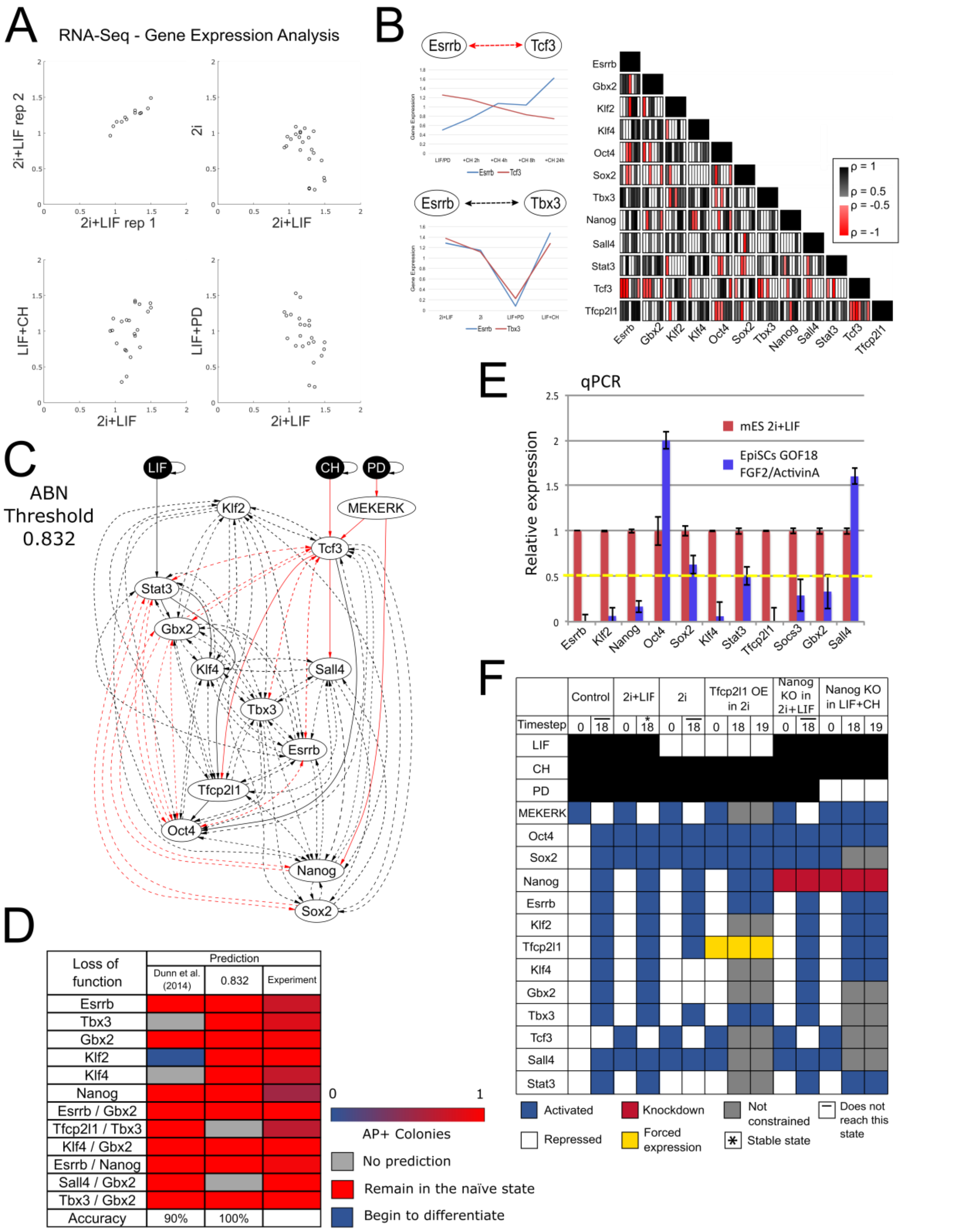

Appendix Figure S1, related to Fig 1: Deriving and constraining the 0.832 ABN. (A) Expression correlation of 12 naive network components, measured by RNA-seq in the indicated culture conditions. RNA-seq data was the average of two technical replicates and strong positive correlation was obtained between replicates (panel top left). The RNA-seq expression is one representative experiment out of seven independent experiments, with the remaining six experimental values measured using qRT-PCR. (B) Left: Examples of gene expression correlation between gene pairs used to infer possible

interactions. Strong negative (Esrrb and Tcf3) and positive (Esrrb and Tbx3) correlations indicate possible negative and positive interactions respectively. Since correlations do not confirm a directed causal relationship, possible interactions are defined to be bidirectional, i.e. Esrrb could negatively regulate Tcf3 and *vice versa*. Right: Summary of Pearson coefficients for each gene pair for all network components based on seven experimentally obtained expression datasets (six qRT-PCR and one RNA-sequencing data). Red: negative coefficient below -0.5. Black: positive coefficient above 0.5. (C) The ABN defined by a Pearson correlation threshold of 0.832. This is constrained by the imposed experimental observations to become the 0.832 cABN (Fig 1B). Required and disallowed interactions have been subsequently identified. (D) Comparing predictions for the 0.832 cABN against the naive state maintenance cABN derived by Dunn et al. (2014). Here, predictions correspond to whether an ESC will remain in the self-renewing state under the indicated knockdown. Mean of n=4 independent experiments. Note an increase in the number of predictions, with previously incorrect predictions correctly made in the 0.832 cABN. (E) Relative gene expression of naïve network components in GOF18 EpiSCs compared to ESCs grown in 2i+LIF. The yellow dashed line indicates the threshold used to discretise expression as High or Low. Only genes significantly above the threshold (Oct4, Sox2 and Sall4) were considered High in EpiSCs. Mean+/-SEM, n=5 independent experiments. (F) Discretisation of gene expression patterns to define the six EpiSC resetting experimental constraints depicted in Fig 1C. Each constraint consists of an initial (timestep 0) and final state (timestep 18), which is either stable (asterisk) or unreachable (bar). Components may be knocked down (red) or under forced expression (yellow). If a specific gene expression is unknown, it is unconstrained (grey).

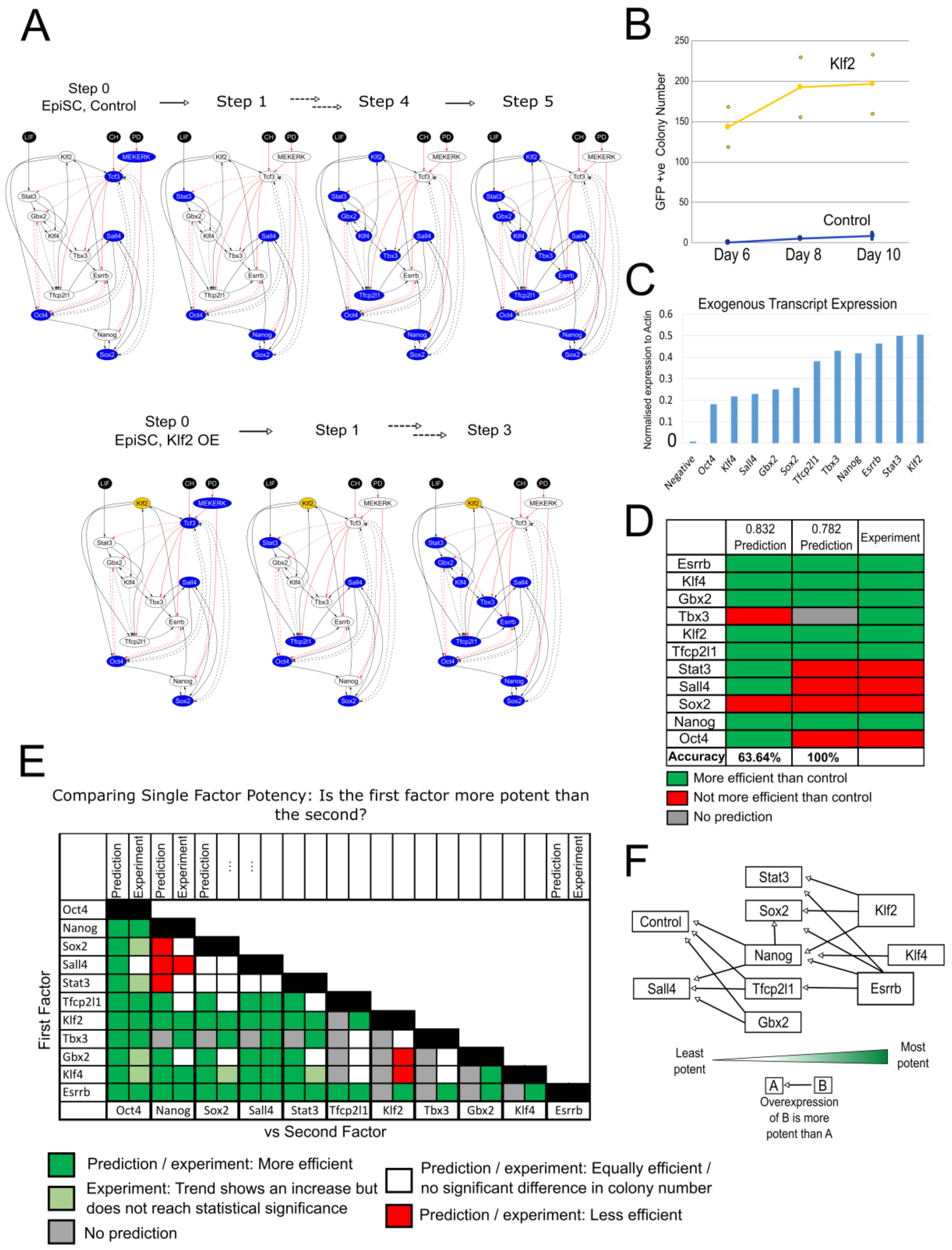

Appendix Figure S2, related to Fig 1: Predicting the relative potency of single factor forced expression in EpiSC resetting. (A) Schematic representation of 0.832 cABN dynamics under forced expression of Klf2, which allows the network to stabilise in the naïve state in fewer steps compared to empty vector control in 2i+LIF (Fig 1B). (B) Oct4-GFP^{+} colony number measured over the resetting time course under empty vector control and forced Klf2 expression. Average colony number of two independent experiments connected with yellow lines, with each independent experiment represented as a dot above and below the average. One representative experiment of two is shown.

(C) Expression of exogenous transcription normalised to actinβ from one representative experiment out of two. (D) A summary of the predictions from the 0.832 and 0.782 cABNs of whether the indicated forced expression was more efficient than empty vector control, compared with experiment. The predictive accuracy of the models increases in the 0.782 cABN. Experimental data was based on data shown in Fig 1E. (E) Comparison summary of predictions and experimental results for resetting potency between gene pairs. Each row compares the prediction from the 0.782 cABN (left box) with experiment (right box), showing whether the first factor (row) is more/less potent (green/red) than the second factor (column). We show experimental results where there was a significant difference between the resulting colony number (Student's t-test, $p<0.05$). In none of the cases tested were the predictions in disagreement with the experimental results (i.e. prediction of gene X being more efficient than gene Y, when in fact gene Y was significantly more efficient than X). The experimental data was based on data shown in Fig 1E. (F) Schematic summary of (E), illustrating the relative potency between individual factors confirmed by experiment, where the arrow points from a more potent to a less potent factor.

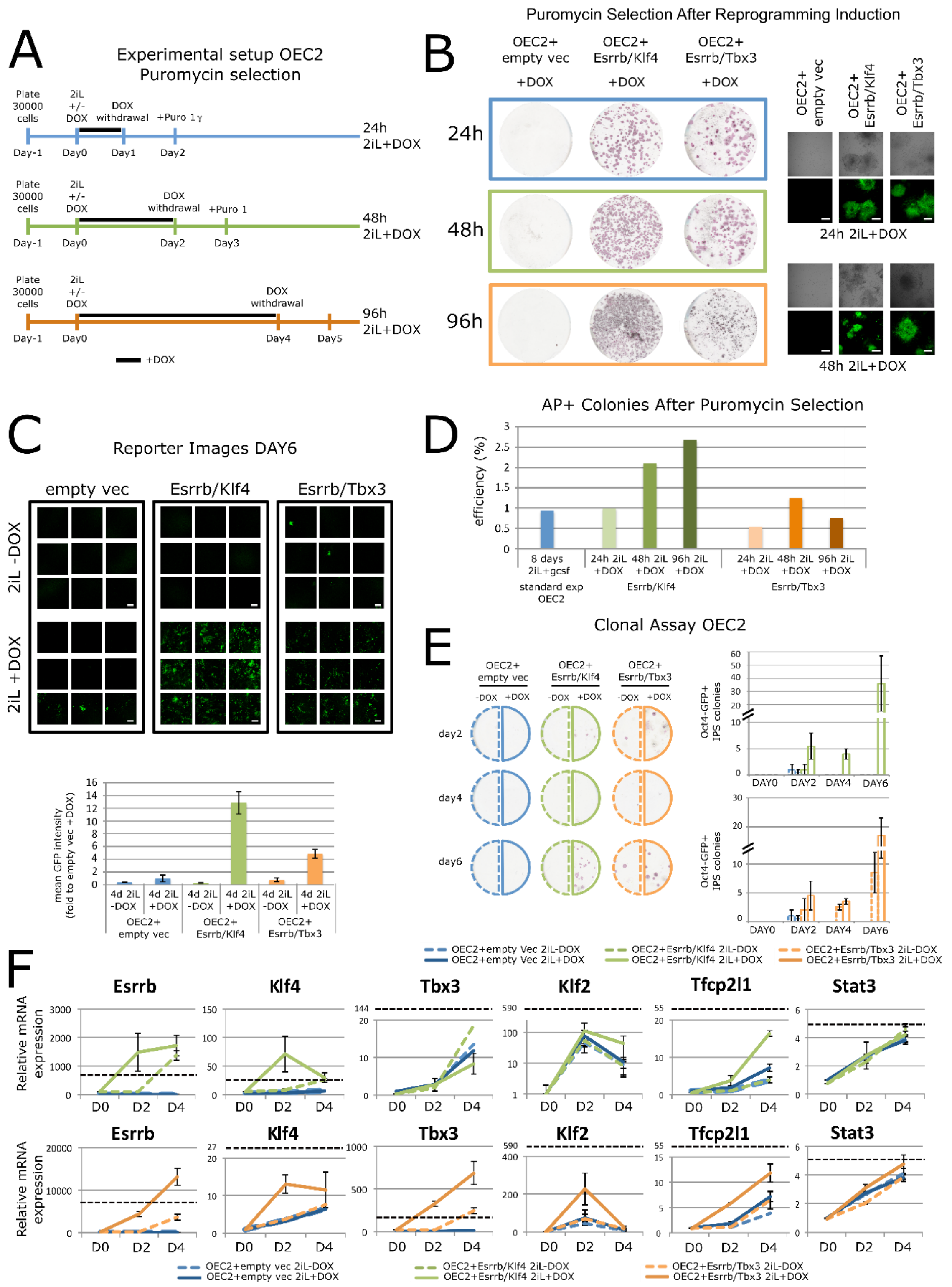

Appendix Figure S3, related to Fig 3: Investigating dual factor expression in OEC2-GY118 EpiSCs. (A) Experimental scheme for the functional characterisation of Esrrb-T2A-Klf4 or Esrrb-T2A-Tbx3 forced expression in OEC2 EpiSC resetting. (B) Left: Representative images of AP staining of reset colonies generated from OEC2-Y118 EpiSCs stably transfected with a *piggyBac* empty vector or vector harbouring Esrrb-T2A-Klf4 or Esrrb-T2A-Tbx3. Cells were treated with 2i+LIF to induce resetting and with DOX for 24h, 48h and 96h (grey, green and orange boxes) to induce the transgene expression. Puromycin selection was applied 24 hours after DOX withdrawal and AP staining performed at Day 8

of resetting. Right: Representative confocal images of Puromycin selected Oct4-GFP$^+$ colonies from 1 or 2 days of DOX treatment. n=2 independent experiments. (C) Representative confocal images (top) and Oct4-GFP mean intensity quantification (bottom) of OEC2-Y118 EpiSCs expressing Esrrb-T2A-Klf4, Esrrb-T2A-Tbx3 or the empty vector control at Day 6 of resetting. Mean +/- SD of n=9 technical replicates. Two independent experiments showed comparable results. (D) Percentage of AP$^+$ colonies in each condition relative to the control treatment (blue), which was in 2i with GCSF for 8 days. Cells expressing Esrrb-T2A-Klf4 (green) or Esrrb-T2A-Tbx3 (orange) were treated with 2i+LIF and DOX for 1, 2 or 4 days, represented by light, medium and dark green respectively. One representative experiment out of two is shown. (E) Left: Representative images of AP staining of colonies generated from resetting OEC2-Y118 EpiSCs stably transfected with an empty vector (blue) or with a *piggyBac* vector containing Esrrb-T2A-Klf4 (green) or Esrrb-T2A-Tbx3 (orange). Cells were treated with 2i+LIF with or without DOX for 2/4/6/8 days (dashed versus solid line), and were subsequently replated at a density of 300 cells/well and cultured for 8 to 10 days in 2i+LIF. Right: Quantification of the number of AP$^+$ colonies generated from clonal assay performed at Day 2/4/6/8 of reprogramming of Esrrb-T2A-Klf4 (top) and Esrrb-T2A-Tbx3 (bottom) expressing cells. Mean +/- SEM, n=2 independent experiments. (F) Relative expression of network components in F/A, at Day 2 and 4 of resetting in the presence or absence of DOX. Black dashed line indicates expression levels in mouse ESCs maintained in 2i+LIF. Gapdh served as an internal control. Mean +/- SEM, n=2 independent experiments. Scale bars in panels (B) and (C) equal 300µm.

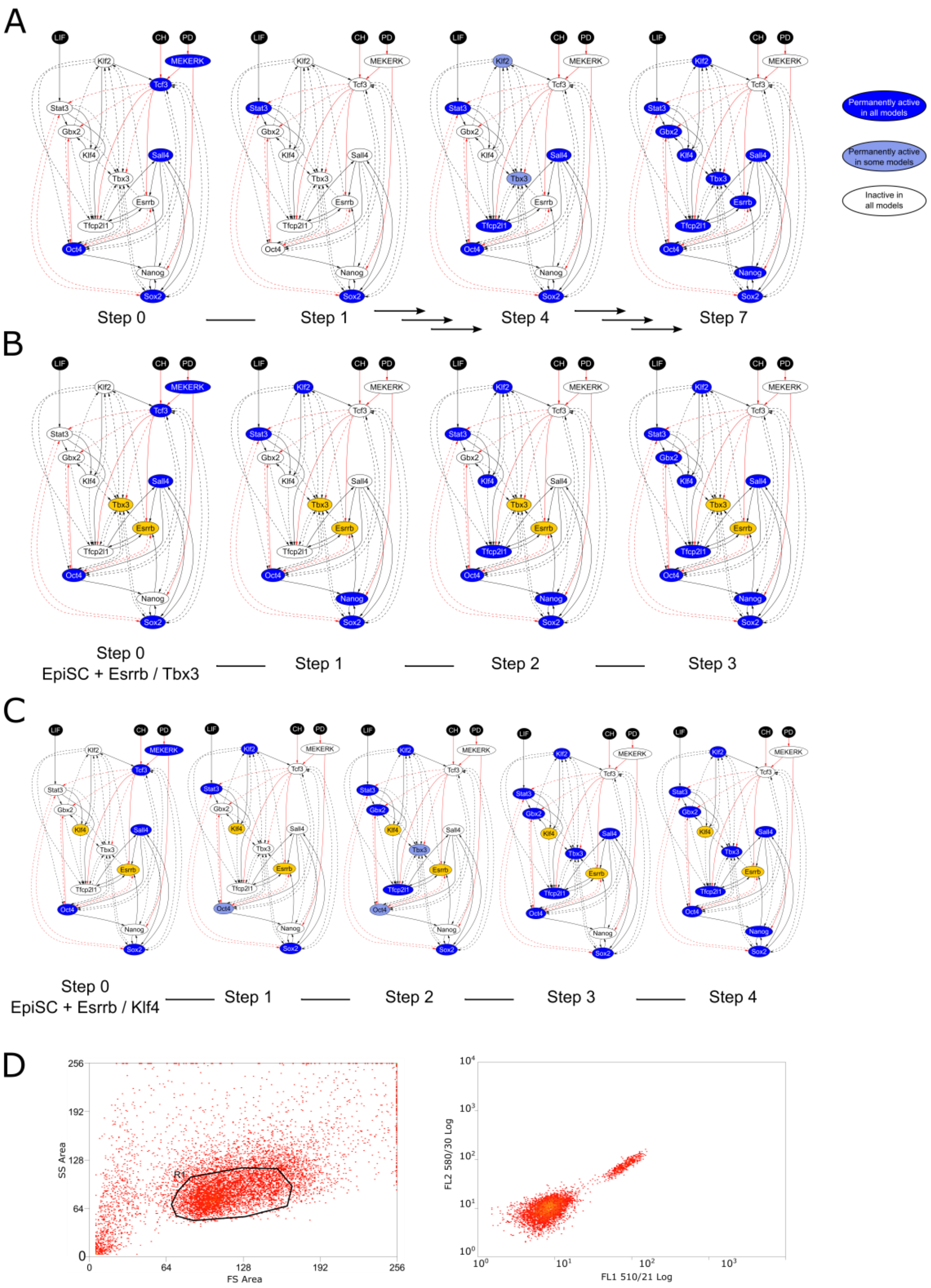

Appendix Figure S4, related to Fig 3, 4a, b: A comparison of the resetting kinetics under empty vector control and dual factor expression, visualised on the 0.782 cABN. (A) Resetting under 2i+LIF alone, which takes 7 steps to stabilise in the naïve state. (B) Resetting under dual expression of Esrrb and Tbx3 in 2i+LIF, which takes 3 steps to stabilise in the naïve state. (C) Resetting under dual expression of Esrrb and Klf4 in 2i+LIF, which takes 4 steps to stabilise in the naïve state. (D) Gating strategy used in the FACS experiments described in Fig 4D. First, individual cells were separated from debris and small cell clusters using Forward Scatter Area vs Side Scatter Area (left). GFP/Venus negative cells were

then identified by examining the level of fluorescence at 510/21 nm using un-induced E/K+EpiSCs as control. Gates used to sort GFP/Venus positive cells are indicated in Fig 4D.

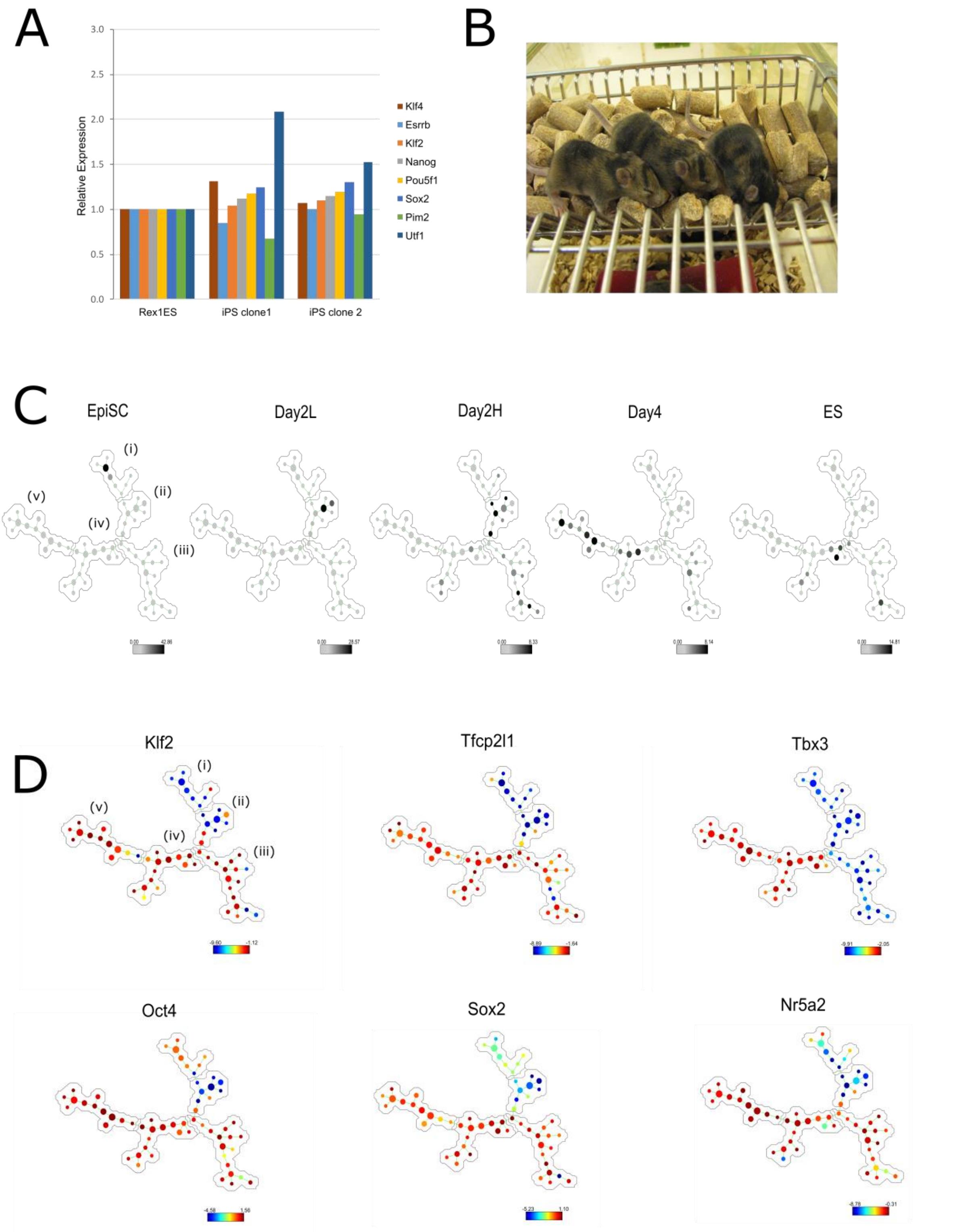

Appendix Figure S5, related to Figs 4 and 5: Analysis of resetting time course using SPADE and clones generated by forced expression of Esrrb-T2A-Klf4 for 4 days. (A) Expression of naïve and early differentiation markers is comparable in Rex1-GFP mESCs (RGd2) and Day4 high cells cultured in 2i+LIF without DOX after 3 passages. Results are from one representative experiment out of two. (B) Contribution to adult chimeras after blastocyst injection of reset clones confirming the naïve pluripotent identity after resetting by Esrrb/Klf4 dual expression. Results are from one representative experiment out of two. (C) Single cell clustering from each time point using the SPADE algorithm. Each

dot represents a group of cells with the size reflecting cell density. Cells from each time point predominantly, but not exclusively, cluster with a branch of the tree, with five populations progress from EpiSCs (branch i) to Day4 high (branch v). (D) SPADE analysis of single cell gene expression of naïve network components along the resetting trajectory, where clusters are coloured according to the expression of the indicated factor.

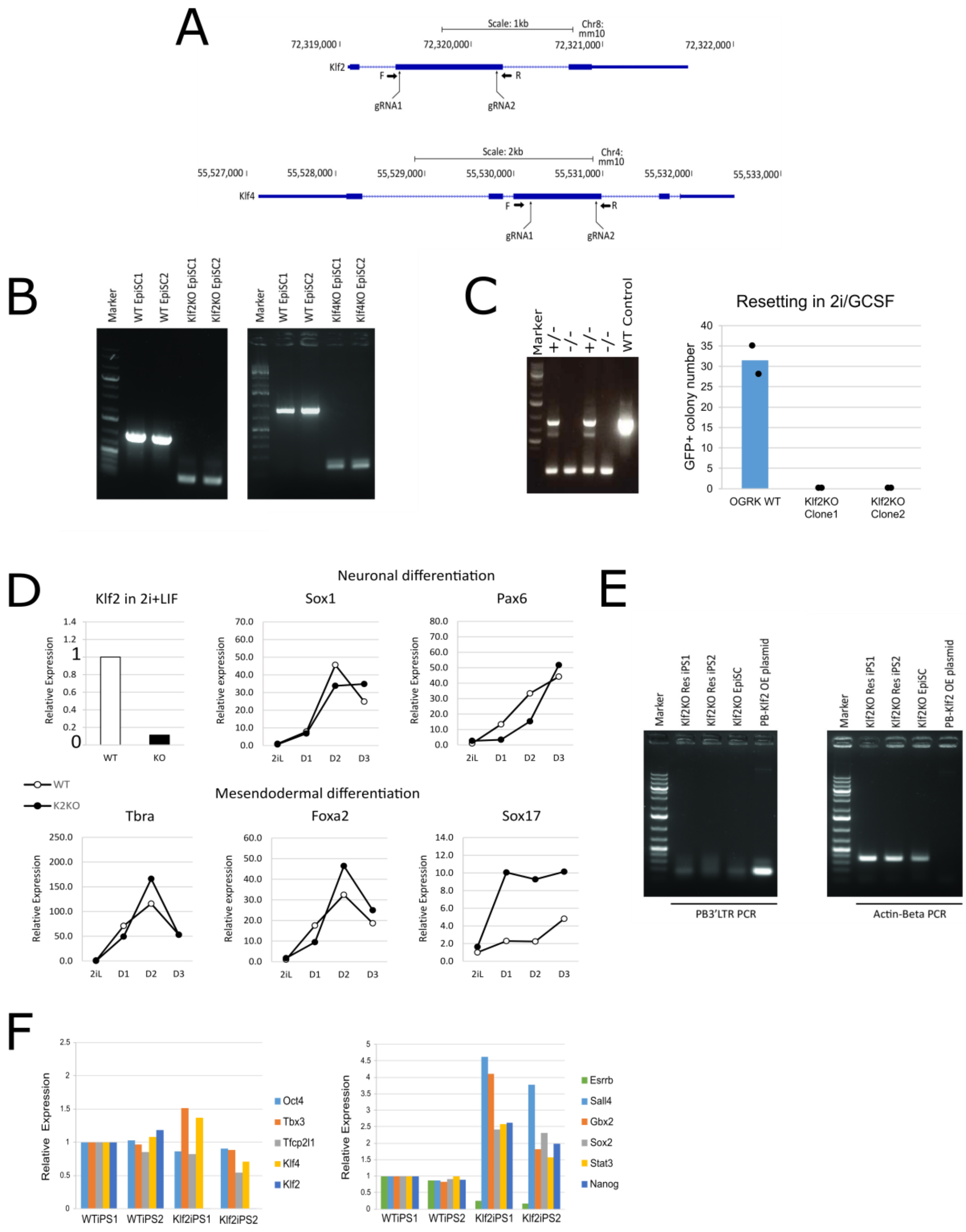

Appendix Figure S6, related to Fig 6: Klf2 and Klf4 KO EpiSC generation and transgene free Klf2 KO iPSCs. (A) Strategies for generating Klf2 and Klf4 KO GOF18 EpiSCs using CRISPR/Cas9. Two guide RNAs were designed to flank the largest coding exons for each gene. (B) Genotyping confirmation for homozygous deletion of Klf2 (left) and Klf4 (right) mutants in two independent clones. (C) We derived a new EpiSC line (OGRK) from E5.5 embryo using N2B27 medium supplemented with ActivinA/Fgf2/Xav939 on Fibronectin, which does not reset spontaneously in 2i+LIF. Stable expression of a chimeric GCSF/LIF GY118F receptor (Yang et al., 2010) allows resetting in the presence of 2i+GCSF.

We generated Klf2 KO OGRK lines with the strategy described in panel (A) (left) and observed that in the absence of Klf2, resetting was abolished (right), as observed in GOF18 EpiSCs. Data points represent two independent experiments. (D) Gene expression of differentiation markers in wild type and Klf2 KO ES cells generated as in (A) from one representative experiment out of two. Cells were exposed to monolayer differentiation protocols for neuroectoderm and mesoendoderm under defined conditions (as described in Mulas et al., 2017) and analyses every 24 hours for 3 days. The values are normalised to wild type ES cells in 2i+LIF and Actinβ is used as the internal control. (E) Klf2 KO reset cells generated by transgene overexpression have not stably integrated the transgene, as demonstrated by the genomic PCR detecting transgene plasmid backbone fragment PB3'LTR (left). Actinβ genomic PCR (right) serves as a PCR control. (F) Gene expression of naïve and transition markers in transgene free iPSCs derived from wild type and Klf2 KO EpiSCs after 3 passages. Results are from one representative experiment out of two.

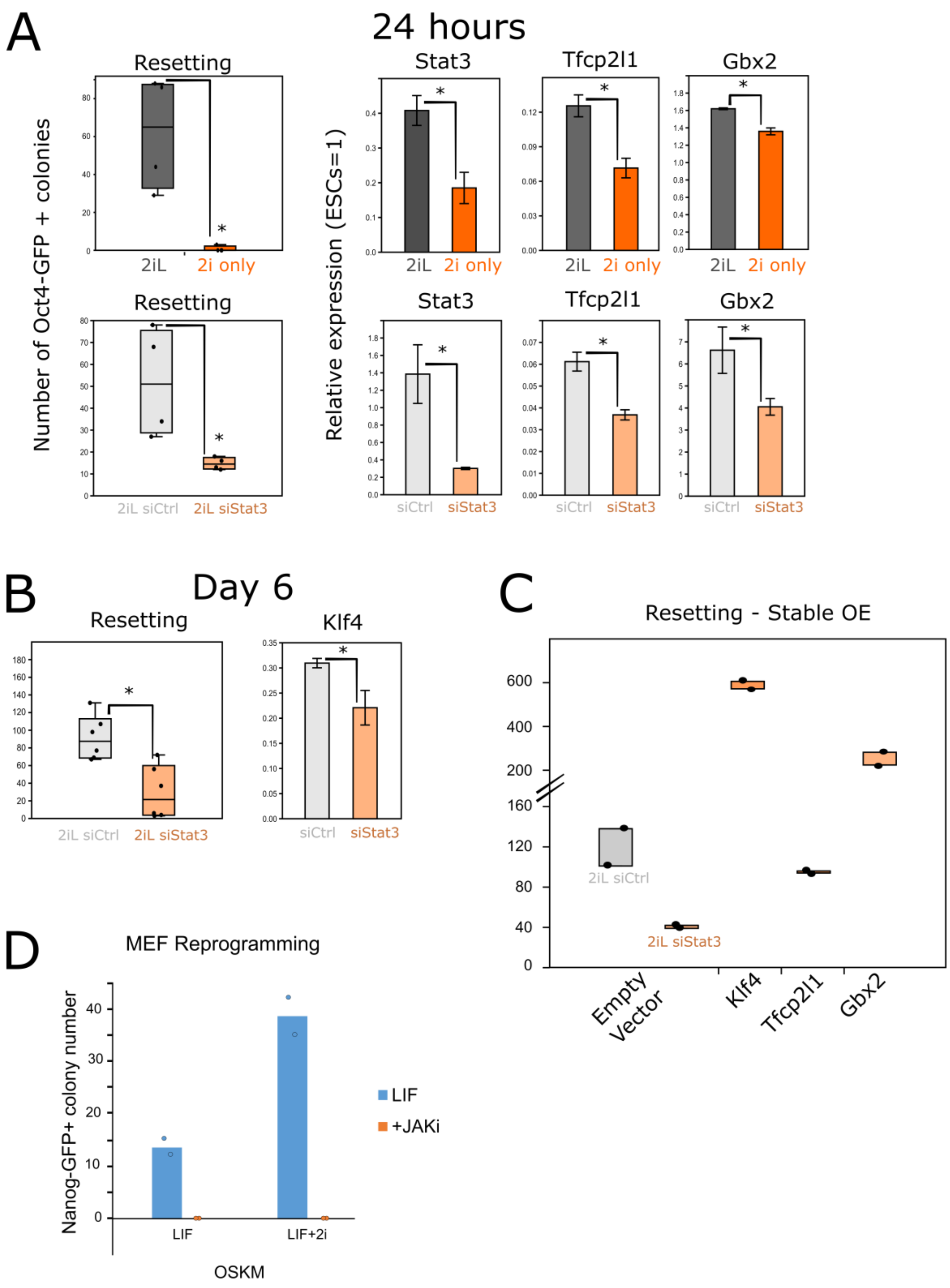

Appendix Figure S7, related to Fig 7: Investigation of Stat3 downstream effectors in EpiSC resetting (A-C, related to Fig 6), and LIF requirement in MEF reprogramming. (A) Left, EpiSC resetting efficiency measured by Oct4-GFP^{+} colony formation in 2i alone, or upon Stat3 knockdown compared to 2i+LIF. n=4 and 3 independent experiments, respectively. Mean+/-SEM. *: p-value<0.05, Student's t-test. Right, induction of Stat3, Tfcp2l1 and Gbx2 expression was impeded in 2i alone, or under Stat3 knockdown in 2i+LIF at 24 hours of EpiSC resetting. n=4 independent experiments, Mean +/-SEM. *: p-value<0.05, Student's t-test. Box-plots indicate 1st, 2nd and 3rd quartile, whiskers show min and max

value. (B) Right: Reduction of Klf4 expression was observed by qPCR at Day 6 of resetting upon Stat3 knockdown. Mean +/-SEM of n=2 independent experiments. Left: quantification of the number of iPSC colonies obtained after Stat3 knockdown. Two independent experiments conducted with 3 technical replicates each. Box-plots indicate 1st, 2nd and 3rd quartile, whiskers show min and max value. (C) Effect of Stat3 knockdown on the Oct4-GFP^{+} colony formation capacity of GOF18 EpiSCs stably overexpressing Tfcp2l1, Gbx2 or Klf4. n=2 independent experiments shown as dots. (D) Nanog-GFP^{+} iPSC formation in OSKM-driven MEF reprogramming in the presence or absence of LIF signalling inhibitor Jaki. Bars indicate mean of n=2 independent experiments, shown as dots.

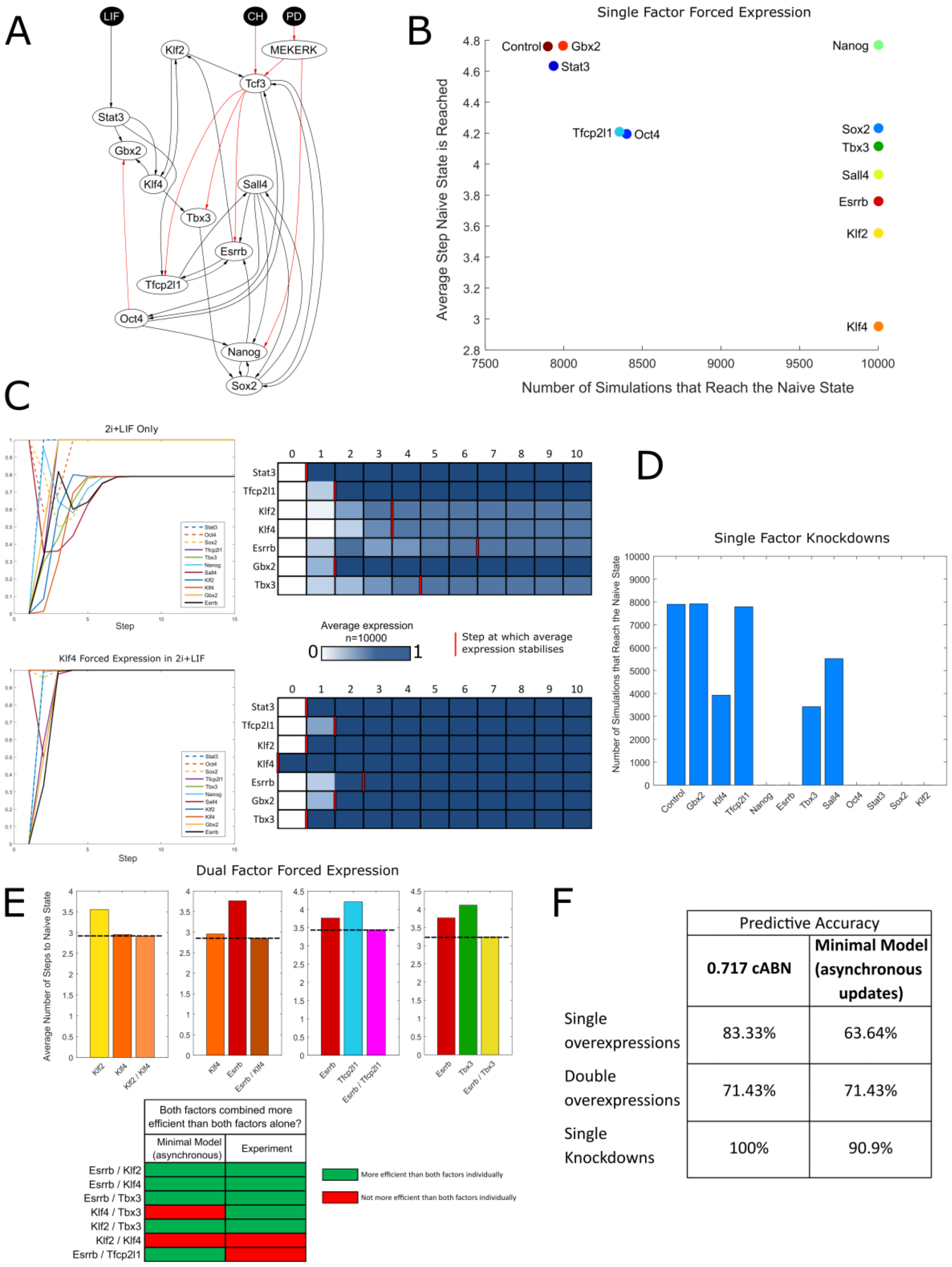

Appendix Figure S8: Simulations of the 0.717 cABN minimal model under asynchronous updates (please see Supplementary Information). (A) The minimal network derived from the 0.717 cABN. (B) Of the 10,000 simulations run under each indicated single forced expression, we calculated the number of simulations that can reach the naïve state and the average number of steps required. See Fig 1E for experimental results of single factor forced expression. (C) Left: simulation trajectories showing the average gene expression of each component at each step during spontaneous resetting. See Fig 2 for relevant experimental results. Right: The gene expression at each time step, averaged

over all simulations. The red border marks the step at which the gene reaches a stable average expression level. (D) Under each of the indicated single knockdowns, we counted the number of simulations that reached the naïve state. Some knockdowns, e.g. Klf2, are predicted to block resetting completely. See Fig 7B for relevant experimental results. (E) We compare the average number of steps required to reach the naïve state under dual factor expression to that required under single factor expression only. Top panels show the same four combinations investigated in Fig 3B. The bottom panel shows a summary of all dual factor expressions investigated in this study. See also Table S3, tab "Resetting Dual Factors" for a summary of all predictions generated under synchronous update schemes and experimental results. (F) A summary of the predictive capacity of the minimal model under asynchronous updates, compared with the complete 0.717 cABN for this set of tests. See also Table S3 for direct comparison between experimental results and predictions generated using the 0.717 cABN.

3) Appendix Supplementary Tables

Appendix Table S1: siRNAs used in this study.

SiRNA	Supplier	Catalogue Number
Klf4 A	Qiagen	SI01083544
Klf4 B	Qiagen	SI001083593
Klf2 A	Qiagen	SI01083530
Klf2 B	Qiagen	SI01083544
Esrrb A	Qiagen	SI02672110
Esrrb B	Qiagen	SI02739569
Tbx3 A	Ambion	AM16708/223884
Tbx3 B	Ambion	AM16708/223885
Stat3 A	Qiagen	SI01435294
Stat3 B	Qiagen	SI01435301
Gbx2 A	Qiagen	SI01010177
Gbx2 B	Qiagen	SI01010170
Oct4 A	Qiagen	SI01385104
Oct4 B	Qiagen	SI01385097
Oct4 C	Qiagen	SI01385090
Oct4 D	Qiagen	SI01385083
Sox2 A	Qiagen	SI04450068
Sox2 B	Qiagen	SI04041842
Sox2 C	Qiagen	SI01429596
Sox2 D	Qiagen	SI01429589
Negative control	Qiagen	1027280

Appendix Table S2: Real-time quantative PCR primers and probes.

Gene	System	Cat. No.	Forward	Reverse
Beta-Actin	SYBR		ACCAGAGGCATACAGGGACA	ACCAGAGGCATACAGGGACA
Gapdh	SYBR		CAGTGATGGCATGGACTGTG	CAATGCATCCTGCACCAC
Nanog	SYBR		AGGGTGTGCTACTGAGATGCTCTG	CAACCACTGGTTTTTCTGCCACCG
Tfcp2l1	SYBR		GGGGACTACTCGGAGCATCT	TTCCGATCAGCTCCCTTG
Esrrb	SYBR		GGCGTTCTTCAAGAGAACCA	CCCACTTTGAGGCATTTCAT
Klf4	SYBR		CACCATGGACCCGGGCGTGGCTGCCAGAAA	TTAGGCTGTTCTTTTCCGGGGCCACGA
Klf2	SYBR		GGTAGTGGCGGGTAAGCTC	AACTGCGGCAAGACCTACAC
Oct4	SYBR		CAGGGTCTCCGATTTGCAT	GCAGCTCAGCCTTAAGAACA
Sox2	SYBR		GAGTGGAAACTTTTGTCCGAGA	GAAGCGTGTACTTATCCTTCTTCAT
Gbx2	SYBR		CTGTAATCCACATCGCTCTCC	ACGGCAAAGCCTTCTTGG
Stat3	SYBR		GTCCTTTTCCACCCAAGTGA	TATCTTGGCCCTTTGGAATG
Sall4	SYBR		GTGTGTAACATATGCGGGCG	TTGTTGGCCCCATGAGTCAT
Tbx3	SYBR		TGTGCCTGTTGGACCATTAGTT	AGCCAGCTCTACTTGAAAGCAT
Pou3f1	SYBR		TTTCTCAAGTGTCCCAAGCC	ACCACCTCCTTCTCCAGTTG
Otx2	SYBR		GACCCGGTACCCAGACATC	GCTCTTCGATTCTTAAACCATACC
Fgf5	SYBR		AAAACCTGGTGCACCCTAGA	CATCACATTCCCGAATTAAGC
Exogenous cDNA	SYBR		GTAATCATGGTCATAGCTGTTTCCT	CCAGGCTTTACACTTTATGCTTC
Foxa2	Taqman	Mm01976556s1		
Pax6	Taqman	Mm00443081m1		
Fkl1	Taqman	Mm01222421_m1		
Sox1	UPL	Probe: #60	GTGACATCTGCCCCCATC	GAGGCCAGTCTGGTGTCAG
Pdgfa	UPL	Probe: #68	GATGAGGACCTGGGCTTG	GATCAACTCCCGGGGTATCT
Tbra	UPL	Probe: #100	CAGCCCACCTACTGGCTCTA	GAGCCTGGGGTGATGGTA
Eomes	UPL	Probe: #9	ACCGGCACCAAACTGAGA	AAGCTCAAGAAAGGAAACA
Sox17	UPL	Probe: #97	CACAACGCAGAGCTAAGCAA	CGCTTCTCTGCCAAGGTC

Appendix Table S3: SDNA oligonucleotides used to generate gRNAs

Klf2 gRNA1 F	CACCGCTCATCCGTGCCGCCCGCCT
Klf2 gRNA1 R	AAACAGGCGGGCGGCACGGATGAGC
Klf2 gRNA2 F	CACCGGGTGTAGGTCTTGCCGCAGT
Klf2 gRNA2 R	AAACACTGCGGCAAGACCTACACCC
Klf4 gRNA1 F	CACCGCACCGTCGCCGCCAGGTCGT
Klf4 gRNA1 R	AAACACGACCTGGCGGCGACGGTGC
Klf4 gRNA2 F	CACCGAGTCGTGTGTGTTGGGCCGG
Klf4 gRNA2 R	AAACCCGGCCCAACACACACGACTC
Klf2F (Genotyping)	AGGGCCTAGTTGTTAGACTTTGG
Klf2R (Genotyping)	GTACGCAGATGCGCCTTTAG
Klf4F (Genotyping)	GTGAGGAACTCTCTCACATGAAGC
Klf4R (Genotyping)	GACTCAGTGTAGGGGTAGTCCTG

Appendix Table S4: Custom Taqman OpenArray real-time quantitative PCR IDs used for the single cell gene expression.

Gene	Taqman ID	House Keeping control
Pou5f1	Mm03053917_g1	
Nanog	Mm02019550_s1	
Sox2	Mm03053810_s1	
Esrrb	Mm00442411_m1	
Nr0b1	Mm00431729_m1	
Tcf7l1	Mm01188711_m1	
Tdgf1	Mm03024051_g1	
Prdm14	Mm01237814_m1	
Dppa3	Mm01184198_g1	
Klf4	Mm00516104_m1	
Klf2	Mm00500486_g1	
Klf5	Mm00456521_m1	
Gbx2	Mm00494578_m1	
Tfcp2l1	Mm00470119_m1	
Tbx3	Mm01195726_m1	
Sall4	Mm00614351_m1	
Nr5a2	Mm00446088_m1	
Lifr	Mm00442942_m1	
Dnmt3L	Mm00457635_m1	
Dnmt3a	Mm00432881_m1	
Dnmt3b	Mm01240113_m1	
Lin28a	Mm00524077_m1	
Tcf15	Mm00493442_m1	
Pou3f1	Mm00843534_s1	
Otx2	Mm00446859_m1	
Pim2	Mm00454579_m1	
Utf1	Mm00447703_g1	
Dppa5a	Mm01171664_g1	
ActB	Mm02619580_g1	Yes
Atp5a1	Mm00431960_m1	Yes
PPIA	Mm02342430_g1	Yes
Tbp	Mm01277042_m1	Yes
Gapdh	Mm99999915_g1	Yes
